# Supplementary material for: Sonic Hedgehog switches on Wnt/planar cell polarity signaling in commissural axon growth cones by reducing levels of Shisa2
Source: eLife. 2017 Sep 8;6:e25269. doi: 10.7554/eLife.25269 (PMC5779225; doi:10.7554/eLife.25269)
Supplement: Transparent reporting form [file elife-25269-transrepform.docx]

***eLife’s* transparent reporting form**

We encourage authors to provide detailed information *within their submission* to facilitate the interpretation and replication of experiments. Authors can upload supporting documentation to indicate the use of appropriate reporting guidelines for health-related research (see [EQUATOR Network](http://www.equator-network.org/%20)), life science research (see the [BioSharing Information Resource](https://biosharing.org/)), or the [ARRIVE guidelines](http://www.plosbiology.org/article/info:doi/10.1371/journal.pbio.1000412) for reporting work involving animal research. Where applicable, authors should refer to any relevant reporting standards documents in this form.

If you have any questions, please consult our Journal Policies and/or contact us: [editorial@elifesciences.org](mailto:editorial@elifesciences.org).

**Sample-size estimation**

- You should state whether an appropriate sample size was computed when the study was being designed
- You should state the statistical method of sample size computation and any required assumptions
- If no explicit power analysis was used, you should describe how you decided what sample (replicate) size (number) to use

Please outline where this information can be found within the submission (e.g., sections or figure legends), or explain why this information doesn’t apply to your submission:

Sample sizes were indicated in the legend of each Figure and Supplementary Figure.

For tissue/cell-based gene expression, Fzd3 modification assays and Fzd3 cell surface localization assay (Figure 1, 3, 4, 6 and Figure 6 – figure supplement 2), the sample size (n ≥ 3) allows us to achieve at least 80% power (standard power) to detect the difference with 95% confidence.

For animal phenotype analysis (Figure 5, 6, 7, Figure 1 – figure supplement 1, Figure 5 – figure supplement 1, Figure 6 – figure supplement 1 and Figure 7 – figure supplement 1), the animals with the same genotypes at the same age (or same plasmid was electroporated) exhibit very similar phenotypes. The sample size (n ≥ 3) should allow us to achieve at least 80% power (standard power) to detect the difference with 95% confidence.

**Replicates**

- You should report how often each experiment was performed
- You should include a definition of biological versus technical replication
- The data obtained should be provided and sufficient information should be provided to indicate the number of independent biological and/or technical replicates
- If you encountered any outliers, you should describe how these were handled
- Criteria for exclusion/inclusion of data should be clearly stated
- High-throughput sequence data should be uploaded before submission, with a private link for reviewers provided (these are available from both GEO and ArrayExpress)

Please outline where this information can be found within the submission (e.g., sections or figure legends), or explain why this information doesn’t apply to your submission:

Sample numbers (n) were indicated in the legend of each Figure and Figure supplement. All graphs were shown by dot/scatter plots with bar graph/error bar (mean ± SD). Sample numbers represents biological replication.

**Statistical reporting**

- Statistical analysis methods should be described and justified
- Raw data should be presented in figures whenever informative to do so (typically when N per group is less than 10)
- For each experiment, you should identify the statistical tests used, exact values of N, definitions of center, methods of multiple test correction, and dispersion and precision measures (e.g., mean, median, SD, SEM, confidence intervals; and, for the major substantive results, a measure of effect size (e.g., Pearson's r, Cohen's d)
- Report exact p-values wherever possible alongside the summary statistics and 95% confidence intervals. These should be reported for all key questions and not only when the p-value is less than 0.05.

Please outline where this information can be found within the submission (e.g., sections or figure legends), or explain why this information doesn’t apply to your submission:

Statistical analysis was used based on the types of data and described in the figure legends and in method, statistics paragraph. One-way ANOVA with post hoc Tukey-Kramer test was performed for multiple comparison, meanwhile student’s *t* test (two-tailed distribution) was performed to compare two groups.

All graphs were shown by dot/scatter plots with bar graph/error bar (mean ± SD).

(For large datasets, or papers with a very large number of statistical tests, you may upload a single table file with tests, Ns, etc., with reference to sections in the manuscript.)

**Group allocation**

- Indicate how samples were allocated into experimental groups (in the case of clinical studies, please specify allocation to treatment method); if randomization was used, please also state if restricted randomization was applied
- Indicate if masking was used during group allocation, data collection and/or data analysis

Please outline where this information can be found within the submission (e.g., sections or figure legends), or explain why this information doesn’t apply to your submission:

Group allocation was indicated in the figure, figure legend and materials and method section (genotypes or transfected plasmids).

**Additional data files (“source data”)**

- We encourage you to upload relevant additional data files, such as numerical data that are represented as a graph in a figure, or as a summary table
- Where provided, these should be in the most useful format, and they can be uploaded as “Source data” files linked to a main figure or table
- Include model definition files including the full list of parameters used
- Include code used for data analysis (e.g., R, MatLab)
- Avoid stating that data files are “available upon request”

Please indicate the figures or tables for which source data files have been provided:

N/A.
